# Impact of supraglottic device with assist ventilation under general anesthesia combined with nerve block in uniportal video-assisted thoracoscopic surgery

**DOI:** 10.1097/MD.0000000000019240

**Published:** 2020-03-06

**Authors:** Xiaobing Xiang, Huidan Zhou, Yingli Wu, Jun Fang, Yanhong Lian

**Affiliations:** a Institute of Cancer and Basic Medicine (ICBM); b Cancer Hospital of the University of Chinese Academy of Sciences; c Zhejiang Cancer Hospital, Hangzhou, Zhejiang, China.

**Keywords:** assist ventilation, intercostal nerve block, paravertebral nerve block, supraglottic device, video-assisted thoracoscopic surgery

## Abstract

**Background:**

With the improvement of anesthesia and surgical techniques, supraglottic device with assist ventilation under general anesthesia (GA) combined with nerve block is gradually applied to video-assisted thoracoscopic surgery. However, the safety of assist ventilation has not been fully confirmed, and a large number of samples should be studied in clinical exploration.

**Methods:**

The subjects included 120 patients, undergoing elective thoracoscopic GA, with American Society of Anesthesiologists (ASA) physical status I or II, were randomly divided into 3 groups, 40 cases in each group. Group T: received double-lumen bronchial intubation, Group I: received intercostal nerve block using a supraglottic device, Group P: received paravertebral nerve block using a supraglottic device. Mean arterial pressure, heart rate, saturation of pulse oximetry and surgical field satisfaction, general anesthetic dosage and recovery time were recorded before induction of GA (T_0_), at the start of the surgical procedure (T_1_), 15 minutes later (T_2_), 30 minutes later (T_3_), and before the end of the surgical procedure (T_4_). Static and dynamic pain rating (NRS) and Ramsay sedation score were recorded 2 hours after surgery (T_5_), 12 hours after surgery (T_6_), 24 hours after surgery (T_7_), time to get out of bed, hospitalization time and cost, patient satisfaction and adverse reactions.

**Results:**

There was no significant difference with the surgical visual field of the 3 groups (*P* *>* .05). The MAP, HR and SpO_2_ of the 3 groups were decreased from T_2_ to T_3_ compared with T_0_(*P* *<* .05). Compared with group T: the total dosage of GA was reduced in group I and group P, the recovery time was shorter, the time to get out of bed was earlier (*P* *<* .05), the hospitalization time was shortened, the hospitalization cost was lower, and the patient satisfaction was higher (*P* *<* .05). The static and dynamic NRS scores were lower from T_5_ to T_7_ (*P* *<* .05). Ramsay sedation scores were higher (*P* < .05), and the incidence of adverse reactions was lower (*P* *<* .05). Comparison between group I and group P: Dynamic NRS score of group P was lower from T_6_ to T_7_ (*P* *<* .05).

**Conclusion:**

Supraglottic device with assist ventilation under general anesthesia combined with nerve block in uniportal video-assisted thoracoscopic surgery is safe and feasible.

## 1 Introduction

Video-assisted thoracoscopic surgery (VATS) has become the mainstream of thoracic surgery, with minimal trauma and is widely recognized. However, general anesthesia with double-lumen bronchial intubation often requires a large number of sedative analgesics and muscle relaxants. Patients have delayed recovery after surgery, and even need ventilator support. Postoperative recovery is slower and complications are more frequent.^[1]^ At present, based on the ERAS concept, it is possible to minimize the perioperative stress reaction, reduce lung injury on the healthy side, and promote early recovery of organ function. Studies have shown that^[2,3]^ general anaesthesia using a supraglottic device with assist ventilation in patients combined with nerve block, making use of artificial atmospheric pressure and automatic collapse of the affected lung, has good effects, fewer complications, and is a more ideal anesthesia.^[4]^ It is one of the important means of implementing rapid rehabilitation surgery in the thoracic department. This study was to observe the feasibility and safety of the application of general anaesthesia using a supraglottic device with assist ventilation in patients combined with nerve block in uniportal video-assisted thoracoscopic surgery, and provide reference for clinical application.

## 2 Subjects and methods

### 2.1 Subjects

#### 2.1.1 Ethics and registration

This study was approved by the Human Research Ethics Board of Zhejiang Cancer Hospital, Hangzhou, with the patient or family members signing of the consent form prior to participation, and the trial was registered with the Chinese Clinical Trial Registry (ChiCTR1900027350).

#### 2.1.2 Inclusion criteria and exclusion criteria

The inclusion criteria were ASA physical status I or II patients, undergoing elective thoracoscopic general anesthesia, aged between 18 and 64 years old, 18 kg/m^2^ < body mass index (BMI) < 24 kg/m^2^, had no history of chest surgery, no history of cardiovascular disease or chronic respiratory disease, no liver, kidney or blood system dysfunction, no history of mental illness. Patients undergoing elective surgeries, including lung wedge, lung lobe, and lung cancer radical surgeries.

#### 2.1.3 Randomization and groups

Using a computer-generated random number table method, subjects were divided into 3 groups, 40 cases in each group. Group T: received double-lumen bronchial intubation; Group I: received intercostal nerve block before induction of the GA using a supraglottic device; Group P: received paravertebral nerve block before induction of the GA using a supraglottic device. All 3 groups were under total intravenous anesthesia. Group T was treated with intubation using muscle relaxant. In group I and group P, muscle relaxants were not used, and assist ventilation was retained using a supraglottic device during surgery.

### 2.2 Methods

#### 2.2.1 Perioperative preparation

On the first day of this study, demographic and medical data, including the patients’ age, weights, and history of diseases were collected. The subjects were not given any sedative or analgesic drugs 24 hours before the operation. Solids and liquids were fasted in patients 8 hours before the operation. On the second day, all selected patients were under monitoring of arterial blood pressure (by way of invasive automated sphygmomanometer), heart rate (by way of electrocardiography), and arterial oxygen saturation (by way of pulse oximetry) before anesthesia induction and after the operation and recovery period, with infusion of compound sodium lactate 200 to 300 ml, through internal jugular vein.

#### 2.2.2 Specific implementation process

Group T received induction of anesthesia with intravenous injection of midazolam 0.05 mg/kg, oxycodone hydrochloride 0.3 mg/kg, propofol 1.0 to 1.5 mg/kg and rocuronium 0.6 mg/kg. After 3 minutes, double lumen bronchus intubation was performed, and mechanical ventilator was administered, V_T_ 6 ml/kg, RR 16 times/minute. During the operation, remifentanil 0.15 to 0.30 μg kg^−1^ min^−1^ and propofol 4 to 8 mg kg^−1^ h^−1^ were maintained via micro-pump, and cisatracurium was intermittently added to maintain muscle relaxation. The EEG bispectral index (BIS) value was between 45 and 60. Patients were helped to adopt a lateral position on the normal side. A 3-cm incision was made between the 4th and 5th intercostal space in the midaxillary line, and the thoracoscope was placed in. After the affected pulmonary collapsed, complete resection of the lesion was done, and the lung was inflated to test for leakage. After confirmation, a thoracic drainage tube was placed in and the chest was closed.

**Figure 1:**
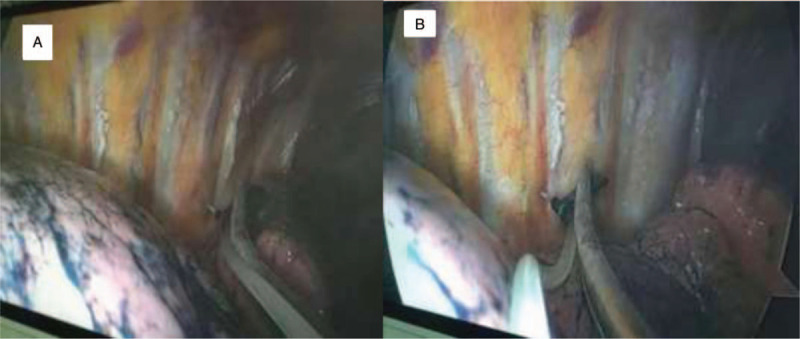
Intercostal nerve block. A. Before intercostal nerve block; B. After intercostal nerve block.

**Figure 2:**
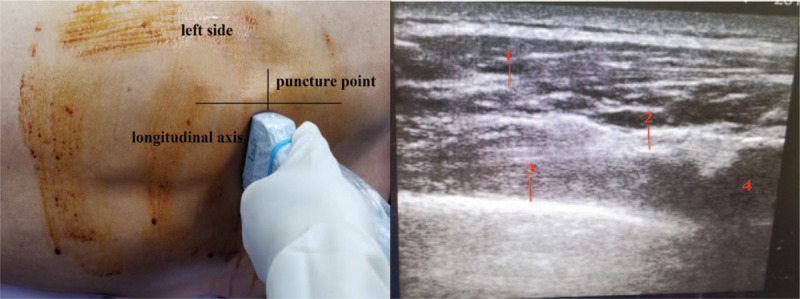
Paravertebral nerve block. 1. Puncture needle; 2. Superior costotransverse ligament; 3. Pleura; 4. T7.

Both group I and group P were injected intravenously with dexmedetomidine 0.4 to 0.8 μg kg^−1^ in 10 minutes, and then received intravenous injection of midazolam 0.02 mg/kg, oxycodone hydrochloride 0.2 mg/kg and propofol 1.5 to 2.0 mg/kg. 2 minutes later, the I-gel supraglottic device was placed in, and patients were connected to the breathing circuit to maintain ventilation. During the operation, dexmedetomidine 0.5 to 1.0 μg kg^−1^ h^−1^, remifentanil 0.03 to 0.06 μg kg^−1^ min^−1^ and propofol 1.5 to 3.0 mg kg^−1^ h^−1^ were maintained via micro-pump, and the BIS value was between 50 and 70. Patients were helped to adopt a lateral position on the normal side. A 3-cm incision was made between the 4th and 5th intercostal space in the midaxillary line, and the thoracoscope was placed in. Group I patients were injected under thoracoscopy with 10 ml of the mixture of 1% lidocaine and 0.375% ropivacaine to the affected vagus nerve and T5–6, T6–7, T7–8 intercostal nerves. 10 ml of 2% lidocaine was sprayed on visceral pleura and lung surface (Fig. 1). Before the induction of anesthesia, group P patients were made a mark at 2-cm above the upper edge of the T7 spinous process. The skin was sterilized and draped routinely. With ultrasound, a 10-cm long, 21st puncture needle was inserted to reach the superior costotransverse ligament of the affected side (longitudinal axis), and paravertebral nerve block was administered with the injection of 15 to 20 ml of 0.375% ropivacaine (Fig. 2). After 10 minutes, the block level of the chest wall of the affected side was measured to reach T4∼T10, and there was no block effect on the contralateral side. Patients were injected under thoracoscopy with 10 ml of the mixture of 1% lidocaine and 0.375% ropivacaine to the affected vagus nerve. 10 ml of 2% lidocaine was sprayed on visceral pleura and lung surface. The 2 groups were observed for 30 seconds. After the lungs were collapsed and the patients vital signs were stable, the lesions were removed, and the lungs were inflated to test for leakage. After confirmation, a thoracic drainage tube was placed in and the chest was closed.

#### 2.2.3 Outcome variables

Make comparisions of the following aspects among the 3 groups, at the time before anesthesia, at the start of the surgery 15 minutes later, 30 minutes later: the mean arterial blood pressure, heart rate, arterial oxygen saturation, including surgical field satisfaction, general anesthetic dose, and recovery time; at the time 2 hours after surgery (T_5_), 12 hours after surgery (T_6_), 24 hours after surgery (T_7_): static and dynamic NRS scores (0: painfree; 1–3: mild pain, does not affect sleep; 4–6: moderate pain; 7–9: severe pain, can not sleep or wake up from pain; 10: sharp pain), Ramsay sedation scores (1: anxious, restless, irritable; 2: cooperative, oriented, quiet; 3: only responsive to instructions; 4: asleep, but quickly responsive to stimulation; 5: asleep, but slowly responsive to stimulation; 6: can not be woken up), including time to get out of bed, hospitalization time and cost, patient satisfaction, and adverse reactions.

## 3 Statistical analysis

Statistical analysis SPSS 22.0 was adopted. A sample size of 40 in each group was determined to be required for a power of 0.90 and an a-value of 0.05. The measurement data were expressed as mean ± standard deviation 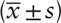, using single factor analysis of variance. The t test was adopted among group comparisons, and the *χ*^*2*^ test was used in enumeration data. If *P* values was < .05, the difference was statistically significant.

## 4 Results

### 4.1 Baseline characteristics

A total of 120 patients were enrolled between March 1, 2019, and August 31, 2019, and the study was finally finished with their data being analyzed for the final results (n = 40 per group). There were no significant statistical differences in general data, operation time, and surgical field satisfaction among the 3 groups (*P* *>* .05) (see Tables 1 and 2).

**Table 1:**
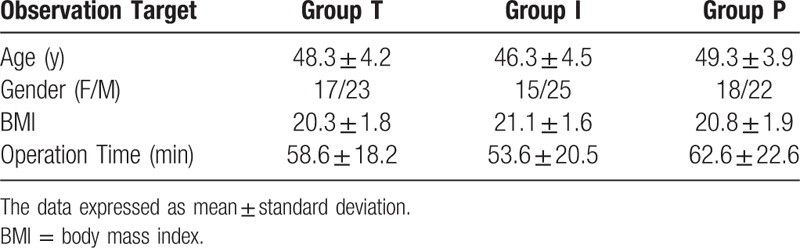
Comparison of clinical data of 3 groups of patients 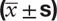.

**Table 2:**
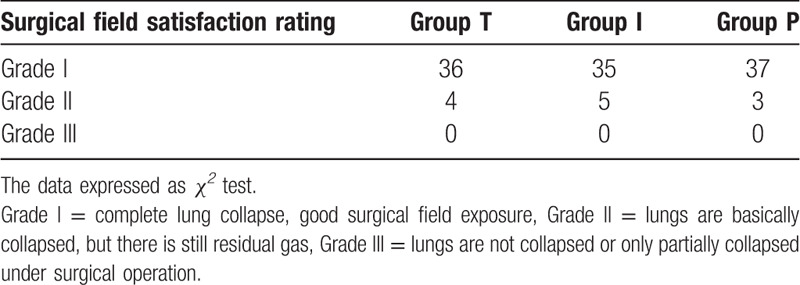
Comparison of surgical field satisfaction of 3 groups (Cases).

### 4.2 Intraoperative variables

The MAP, HR and SpO_2_ of the 3 groups at T_2_∼T_3_ were lower than that at T_0_ (*P* < .05), and there was no significant statistical difference among the other time points (*P* > .05) (see Table 3).

**Table 3:**
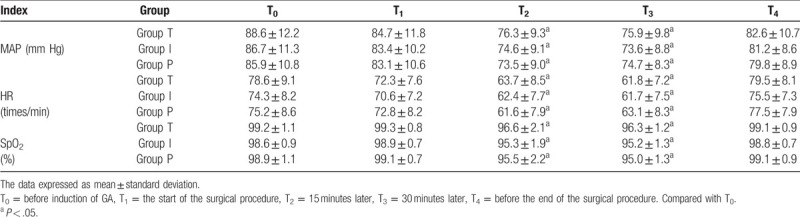
Comparison of MAP, HR, and SpO_2_ of 3 groups at each time point 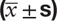.

Compared with group T: the total dose of general anesthesia was reduced in group I and group P, the recovery time was shortened, the time to get out of bed was earlier (*P* < .05), the hospitalization time was shortened, the hospitalization cost was lower, and the satisfaction was higher (*P* < .05). (see Table 4).

**Table 4:**
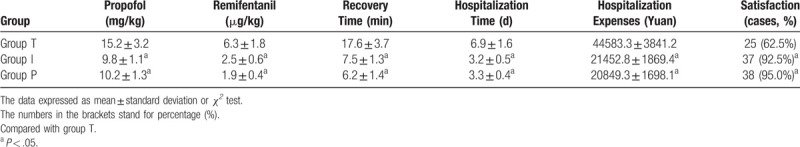
Comparison of intraoperative total anesthetic dose and postoperative status among 3 groups.

Compared with group T: static and dynamic NRS scores of group I and group P were significantly decreased at T_5_∼T_7_ (*P* < .05) and Ramsay sedation scores were increased (*P* < .05); Comparison between group I and group P: dynamic NRS scores of group P were lower at T_6_∼T_7_ (*P* < .05) (see Table 5).

**Table 5:**
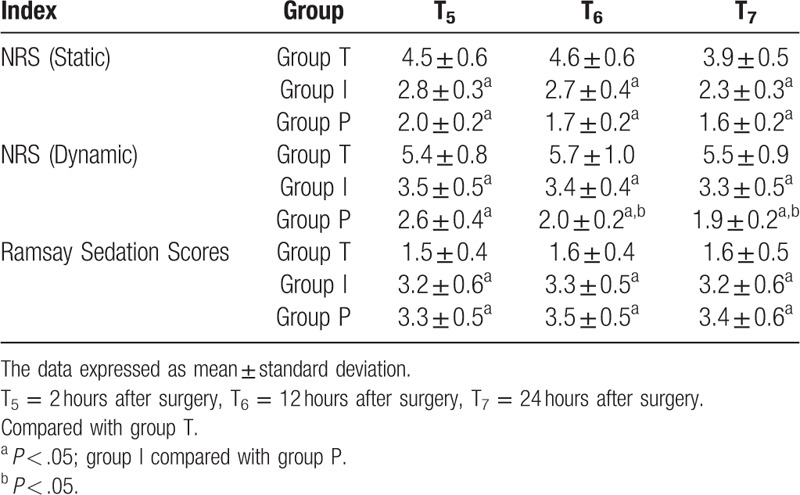
Comparisons of NRS and Ramsay sedation scores among 3 groups (points, 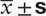).

Compared with group T: the incidence of adverse reactions in group I and group P was lower (*P* < .05) (see Table 6).

**Table 6:**
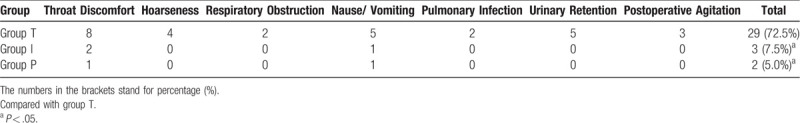
Comparison of adverse reactions (cases (%)).

## 5 Discussion

In the traditional treatment of thoracoscopic surgery, double-lumen bronchial catheter is the most commonly used anesthesia for lung isolation.^[5]^ However, studies have shown that double-lumen bronchial catheters are prone to different degrees of airway and lung injury,^[6,7]^ as well as complications caused by residual muscle relaxants after using muscle relaxants,^[8]^ affecting the postoperative treatment effect. In recent years, some scholars have successively explored the application of general anesthesia (GA) using a supraglottic device with assist ventilation in patients combined with nerve block in thoracoscopic surgery. It is in line with the rapid perioperative recovery concept to help patients maintain ventilation in the natural state, reducing the damage to the individual organ function of the patient and accelerating the healing process.^[9,10]^

It is currently believed that the application of GA using a supraglottic device with assist ventilation in patients combined with nerve block can effectively reduce the damage to the airway, and can significantly increase the safety of the airway. If necessary, it can be applied in positive pressure ventilation to ensure the surgical efficacy.^[11,12]^ Therefore, this study designed 3 different anesthesia programs and observed their effects.

The supraglottic device is the airway device on the glottis. It does not enter the trachea, avoiding mechanical damage to the vocal cords and tracheal mucosa, reducing postoperative throat discomfort, vocal cord damage, etc.^[2]^ Most thoracic surgeries adopt the lateral position on the normal side, and the traditional standard supraglottic device is prone to displacement and leakage. In this study, the I-gel supraglottic device was used, which has a good fixation, high success rate of first insertion, reducing perioperative position changes and possibility of supraglottic device displacement, reducing airway leakage, promoting easier airway management.^[13]^

This study showed that under thoracoscopy, spraying 2% lidocaine on the pleura and lung surface can reduce the surface tension of the lung, which is conducive to lung collapse, and can block the vagus nerve in the ipsilateral thoracic cavity, which is conducive to inhibiting the lung traction reaction and bucking reflexes to keep clear surgical field.

Results of this study showed that the hemodynamics of the 3 groups all at different levels 15 to 30 minutes after the surgery began, but it was basically stable within the normal range. There was no statistically significant difference between the 3 groups, indicating that if the anesthesia effect was perfect and after sedation and analgesia treatment, there would be some degree of hemodynamic fluctuations. Compared with group T, the mediastinal oscillations in patients of group I and group P with assist ventilation did not increase the hemodynamic changes. On the one hand, it may be related to the lung compensation and the change of pulmonary ventilation/blood flow on the ventilated side. On the other hand, the ipsilateral thoracic vagus nerve block reduces the patient's mediastinal oscillation.

This study showed that because of the need for deep anesthesia and the use of muscle relaxants in group T, the doses of propofol and remifentanil in group I and group P were significantly reduced, the recovery time and extubation time were significantly shortened, and there was no risk of residual muscle relaxant. It is conducive to early respiratory function and gastrointestinal function recovery and earlier postoperative activities, reducing patient hospitalization time and expenses, improving patient satisfaction, and increasing the utilization of medical resources.^[14,15]^ During the use of supraglottic device, inhaled anesthetics have the risk of leakage, so intravenous anesthetics are used throughout the anesthesia process in this study, which is different from other scholars’ research.^[16]^

This study used NRS scores to evaluate postoperative analgesia effect. It has shown that the pain is significantly relieved within 24 hours after surgery after the intercostal nerve block and paravertebral nerve block are administered respectively in group I and group P, indicating that nerve block can relieve postoperative incision pain and improve pain tolerance, which is consistent with that in Mogahed research.^[16]^ At the same time, the Ramsay sedation scores of group I and group P increased, which may be related to the reduction of pain by reason of nerve block, bringing indirect sedation. The dynamic NRS score was even lower in group P from 12 to 24 hours after operation, which may be related to the fact that local anesthetic went along the paravertebral space, got through the intervertebral foramen and then reached the epidural space, resulting in multisegment motor nerve block. It also showed that under ultrasound guidance, the nerve block effect was exact and prolonged the analgesic effect.^[17,18]^

Because the supraglottic device is less irritating to the throat and airway, and there were fewer opioids and sedative drugs used in the operation, and no muscle relaxant was used, therefore, the incidence of nausea and vomiting and urinary retention is lower in group I and group P, with fewer adverse reactions such as pharyngeal discomfort, and with rapid recovery of organ function.^[19]^ In addition, there was no pulmonary infection after operation in group I and group P, which further explained that the supraglottic device was safe under assist ventilation.

In summary, the application of general anesthesia using a supraglottic device with assist ventilation in patients combined with nerve block for clinical application in uniportal video-assisted thoracoscopic surgeries is safe and reliable, worth promoting. However, the surgeons and the anesthesiologists need to cooperate well to ensure safe operation and achieve minimally invasive surgery. Meanwhile, the number of samples in this study is limited, therefore, further research on safety can be done.

## Author contributions

XXX.

Xiaobing Xiang orcid: 0000-0002-8026-0106.
